# Prediction of Military Vehicle’s Drawbar Pull Based on an Improved Relevance Vector Machine and Real Vehicle Tests

**DOI:** 10.3390/s16030351

**Published:** 2016-03-10

**Authors:** Fan Yang, Wei Sun, Guoyu Lin, Weigong Zhang

**Affiliations:** 1 Instrument and Meter Engineering, Southeast University, Nanjing 210096, China; 230109465@seu.edu.cn (F.Y.); zhangwg@seu.edu.cn (W.Z.); 2 The 14th Research Institute, China Electronics Technology Group Corporation, Nanjing 210013, China; sunwei0011@sina.com (W.S.)

**Keywords:** relevance vector machine, multiple kernels, particle swarm optimization, drawbar pull, real vehicle test

## Abstract

The scientific and effective prediction of drawbar pull is of great importance in the evaluation of military vehicle trafficability. Nevertheless, the existing prediction models have demonstrated lots of inherent limitations. In this framework, a multiple-kernel relevance vector machine model (MkRVM) including Gaussian kernel and polynomial kernel is proposed to predict drawbar pull. Nonlinear decreasing inertia weight particle swarm optimization (NDIWPSO) is employed for parameter optimization. As the relations between drawbar pull and its influencing factors have not been tested on real vehicles, a series of experimental analyses based on real vehicle test data are done to confirm the effective influencing factors. A dynamic testing system is applied to conduct field tests and gain required test data. Gaussian kernel RVM, polynomial kernel RVM, support vector machine (SVM) and generalized regression neural network (GRNN) are also used to compare with the MkRVM model. The results indicate that the MkRVM model is a preferable model in this case. Finally, the proposed novel model is compared to the traditional prediction model of drawbar pull. The results show that the MkRVM model significantly improves the prediction accuracy. A great potential of improved RVM is indicated in further research of wheel-soil interactions.

## 1. Introduction

The current study intends to predict military vehicles’ drawbar pull utilizing improved relevance vector machine (RVM) for evaluating vehicle trafficability. Vehicle trafficability is considered as one of the most important mobility indexes. Especially for military vehicles, good vehicle trafficability has great significance in guaranteeing the transport capability on the battlefield. Due to the adverse field conditions, military vehicles may suffer from the non-geometric hazards, such as mud, bog, clay, sand and snow, in addition to unknown obstacles. The low cohesion and weak bearing ability of terrains tend to make the wheels get stuck, which causes power consumption or even mission failure. Hence, it is crucial to establish effective evaluation models of vehicle trafficability. Among the various evaluation indexes, such as drawbar pull (Dp), pull coefficient and tractive efficiency, Dp is imposed as a priority in this study [1].

For predicting drawbar pull, the considerable progress made over the last decades can be classified to two categories: semi-empirical models and analytical models [2]. The semi-empirical model was pioneered by Bekker [3,4] and later by Wong [5] to predict the tractive performance of vehicles. This model utilizes two analog devices to represent the wheel-soil interactions. Vertical deformation of the soil under load is assumed analogous to soil deformation under a flat plate while the shear deformation of the soil under a traction force is assumed to be equal to the shear caused by a rectangular grouser unit. The assumptions indeed help to simplify the model and make the model realizable. However, the estimation accuracy is greatly affected with the prediction result inconsistent with the reality. The analytical model is appealing and has developed in recent years, such as finite element modes [6] and discrete element models [7]. It reassures a certain level of understanding of the basis process of wheel-soil interactions. Nevertheless, the complexity of the model and rigorous requirements for numerous soil parameters are the main hindrance in the wide usage of this technique. In summary, no general solution has been obtained to effectively predict drawbar pull [2]. Complex and nonlinear relationships involved inwheel-soil interactions hold it back from expressing and modeling the exact interactions,which is the root cause of prediction errors.

Considering the characteristics of wheel-soil interactions, mathematical statistics methods are suggested as a potential solution for the addressed issue. Without the knowledge of exact structure and inner parameters of models, the target vector can be well predicted from the inputs. Various effective mathematical statistics methods have been developed and successfully used in engineering problems, such as artificial neural network (ANN) [8,9,10], generalized regression neural network (GRNN) [11,12], support vector machine (SVM) [13,14] and relevance vector machine (RVM) [15,16]. Among the methods, a proper method should be picked out to fit the concrete problems. Firstly, the model to be built in this study is a complex nonlinear issue, which is caused by two nonlinear systems, *i.e.*, the vehicle system and terrain system. Secondly, the training set points to a small-sample problem and up to about 100 data points can be provided. Thirdly, due to the limitations of real vehicle test conditions, incomplete test data can be gained. Therefore, RVM are employed as an ideal method due to its applicability to small-sample problems and generalization ability. In recent years, modifications have been done to original RVM methods for better performance [17,18,19]. In this study, a novel prediction model is proposed based on an improved RVM. Multiple kernels are combined to improve its regression performance and nonlinear decreasinginertia weight particle swarm optimization is applied for parameter optimization.

Before establishing the improved RVM model, it is of great importance to confirm the effective influencing factors of drawbar pull. It is indicated from the existing models that drawbar pull can be influenced by wheel diameter/width, inflation pressure (Ip), moving velocity (V), vertical load (W), wheel slip ratio (s), passing times and soil conditions [1]. Nevertheless, it is a tough task to take a whole consideration of all the influencing factors at the present stage. Considering that this study is intended for a better evaluation of military vehicles’ trafficability and to provide instructions for the usage of military vehicles before specific missions, the focus is laid upon the factors, *i.e.*, moving velocity, vertical load, inflation pressure and wheel slip ratio. For the relations between drawbar pull and these four factors, a series of conclusions have been gained by former researchers [20,21,22,23]. The results are obtained by indoor experimental platforms. However, real vehicle conditions demonstrate distinct characteristics, especially the dynamic responses. The exact relations need to be further validated by real vehicle test data.

In this study, another research emphasis is laid on the experimental analysis of drawbar pull’s influencing factors. A dynamic testing system is applied to conduct designed real vehicle tests and gather the dynamic responses of wheel-soil interactions. On the basis of real vehicle test data, we can investigate the effectiveness of drawbar pull’s influencing factors in practice. Then, the proposed RVM model can be established and tested.

## 2. Experimental Data Acquisition and Analysis

In this section, we first make a brief description of the adopted dynamic testing system. Then we discuss the detailed test procedures. Finally, a series of experimental analyses follow to confirm the effective influencing factors of drawbar pull for new modeling.

### 2.1. Dynamic Testing System

**Figure 1:**
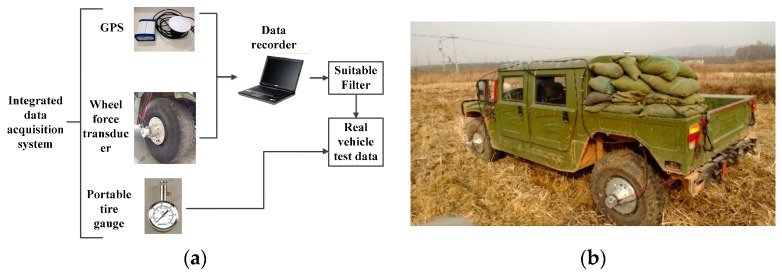
(**a**) Integrated data acquisition system; (**b**) Instrumented test vehicle.

The dynamic testing system consists of the wheel force transducer (WFT), GPS and portable tire gauge as shown in Figure 1a [24]. An integrated data acquisition system is set up to gain test data synchronously and real-time. Thus, an instrumented test vehicle equipped with the dynamic testing system makes it possible to conduct field tests as shown in Figure 1b. Among the primary sensors, the WFT is employed to measure the dynamic responses of drawbar pull, vertical load and wheel angular speed (ω) in wheel-soil interactions; the GPS is prepared for vehicle velocity; the portabletire gauge measures the inflation pressure. And wheel slip ratio can be calculated with ω and V.

### 2.2. Test Procedures

Field tests were conducted in order to gain the essential data for the study of predicting a military vehicle’s drawbar pull. As emphasis is placed on the four influencing factors (moving velocity, vertical load, inflation pressure and wheel slip ratio), the field tests were conducted with five different vertical load of 9, 10, 11, 12 and 13 kN, at three levels of inflation pressure (140, 240 and 340 kPa). A wide range of slip ratio and moving velocity should also be guaranteed, so amongst all the driving maneuvers, the straight accelerate-brake driving was adopted, which provided the most desirable range of data. Summary of the adopted levels of influencing factors is shown in Table 1. A 3.6-ton weight all-wheel-drive military vehicle was used in the test and the static vertical load on the left rear wheel was about 8 kN. The data presented in this study is based on the field tests conducted at the Dingyuan Automotive Proving Ground in An’hui Province, China on 11 December 2014. Kinds of terrains were selected for the tests, such as sandy soil, clayed soil and sandy loam. The presented test data is mainly from the left rear wheel on clayed soil. The detailed experimental proceduresare described as follows. (1) Before real vehicle tests, the soil was collected and transported back to the soil test laboratory for the analysis of semi-empirical model related parameters; (2) It took several minutes to confirm the GPS signal. Then all the other devices were turned on and made sure they were working in normal condition; (3) The vehicle equipped with the dynamic testing system was tested along the planned route and the dynamic responses were recorded by the data acquisition system; (4) After each travel, the level of vertical load and inflation pressure were adjusted. Inflation pressure was measured and controlled by the portable tire gauge. Sandbags were used to provide additional vertical load.

### 2.3. Preliminary Experimental Data Analysis

Raw test data gathered were pre-processed to meet the demand of experimental analysis. Wavelet filter was used here to reduce the high frequency noise while the Savitzky-Golay method was used to smooth the data curve. Based on the data curves of the drawbar pull *versus* time, a Matlab GUI program was developed to draw the curves of the drawbar pull *versus* slip ratio. Then the required data set (V, W, Ip, s, Dp) can be gained. For an investigation of the factors’ effectiveness on drawbar pull, a series of experimental analyses are done separately.

#### 2.3.1. Effect of Velocity on Drawbar Pull

**Figure 2:**
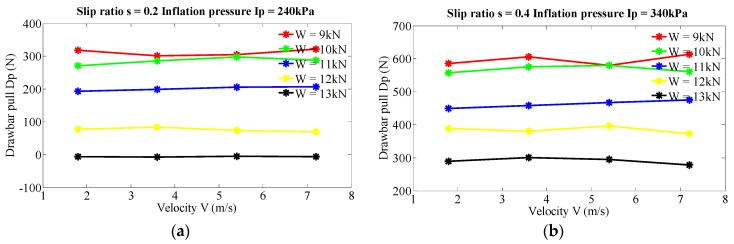
Effect of velocity on drawbar pull at various levels of vertical load, inflation pressure and slip ratio. (**a**) Slip ratio s = 0.2, inflation pressure Ip = 240 kPa; (**b**) Slip ratio s = 0.4, inflation pressure Ip = 340 kPa.

The obtained data were processed for four levels of moving velocity, five levels of vertical load, three levels of inflation pressure and six levels of slip ratio in order to determine the effect of velocity on drawbar pull. Figure 2 illustrates velocity’s effect on drawbar pull at five different vertical loads of 9, 10, 11, 12 and 13 kN, at two couples of constant inflation pressure and slip ratio, (240 kPa, 0.2) in Figure 2a and (340 kPa, 0.4) in Figure 2b. It’s worth pointing out that Figure 2 is shown as a representative of all experimental results. It is inferred that there are no significant differences of drawbar pull when the velocities varying in the range of 0–7.2 m/s. The values of drawbar pull change slightly with no regular pattern, which is probably caused by the measurement uncertainty during field tests. For higher velocities, it is not in the scope of present study due to a limited moving distance on the test field, which is a focus of future research. So, it comes to the conclusion that the effect of relatively low velocity (up to 7.2 m/s) on drawbar pull for the test military vehicle can be ignored. In the following analysis of other factors, velocity’s effect will not be included.

#### 2.3.2. Effect of Vertical Load on Drawbar Pull

**Figure 3:**
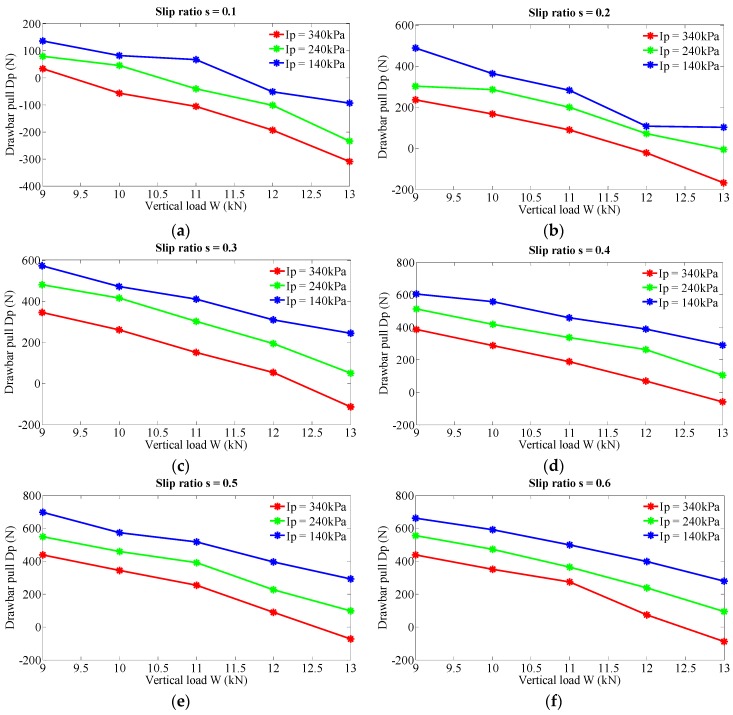
Effect of vertical load on drawbar pull at various levels of inflation pressure and slip ratio. (**a**) Slip ratio s = 0.1; (**b**) Slip ratio s = 0.2; (**c**) Slip ratio s = 0.3; (**d**) Slip ratio s = 0.4; (**e**) Slip ratio s = 0.5; (**f**) Slip ratio s = 0.6.

**Figure 4:**
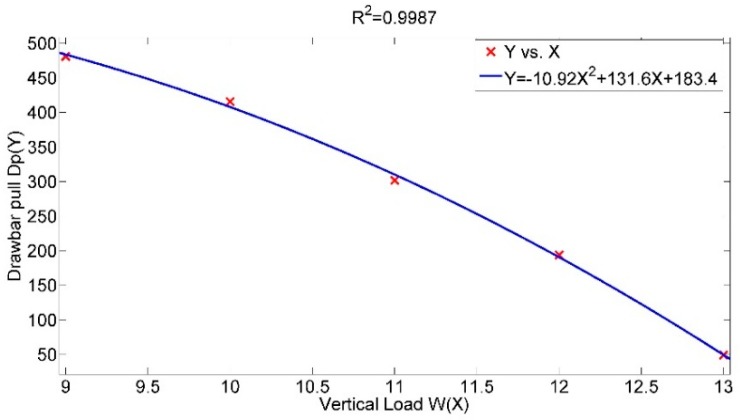
Drawbar pull with respect to vertical load.

To investigate the impact of vertical load on drawbar pull, three levels of inflation pressure (140, 240 and 340 kPa) and six levels of slip ratio (0.1, 0.2, 0.3, 0.4, 0.5 and 0.6) are taken into consideration. Figure 3 shows the investigation results. With an increase of the vertical load, the values of the drawbar pull decrease at all the couples of constant inflation pressure and slip ratio. In Figure 3a, if the slip ratio is set to 0.1 and the inflation pressure 240 kPa, the corresponding drawbar pull decreases from 79.72 N to −234.5 N when the vertical load increases from 9 to 13 kN. The relative increase rate of the vertical load is 44.44% with the resulting changing rate of the drawbar pull as 384.5%. Further numerical analysis indicates that the relation between vertical load and drawbar pull tends to be a quadratic regression relationship rather than a linear relationship as shown in Figure 4 (slip ratio is set to 0.3 and inflation pressure 240 kPa). In general, the variance of vertical load has great effect on drawbar pull and a relatively complicated relation is inferred.

#### 2.3.3. Effect of Inflation Pressure on Drawbar Pull

**Figure 5:**
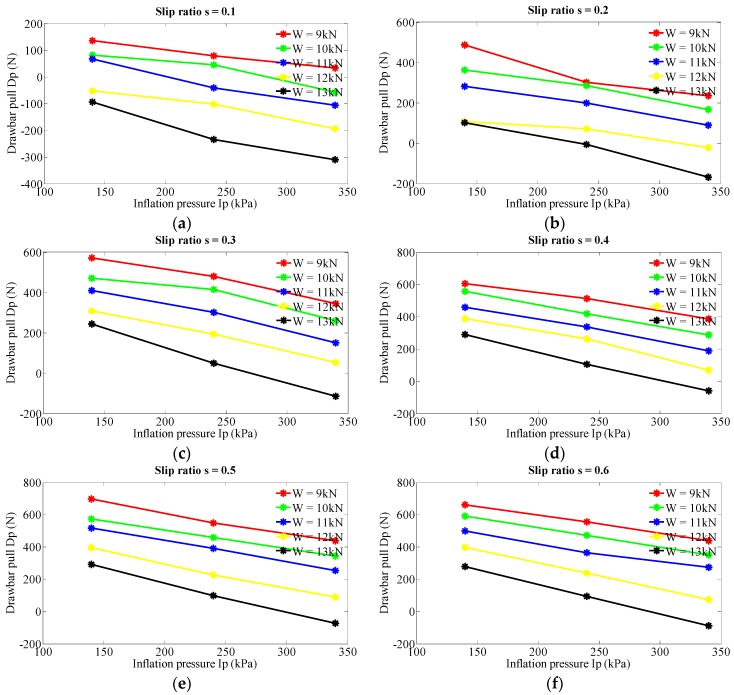
Effect of inflation pressure on drawbar pull at various levels of vertical load and slip ratio. (**a**) Slip ratio s = 0.1; (**b**) Slip ratio s = 0.2; (**c**) Slip ratio s = 0.3; (**d**) Slip ratio s = 0.4; (**e**) Slip ratio s = 0.5; (**f**) Slip ratio s = 0.6.

**Figure 6:**
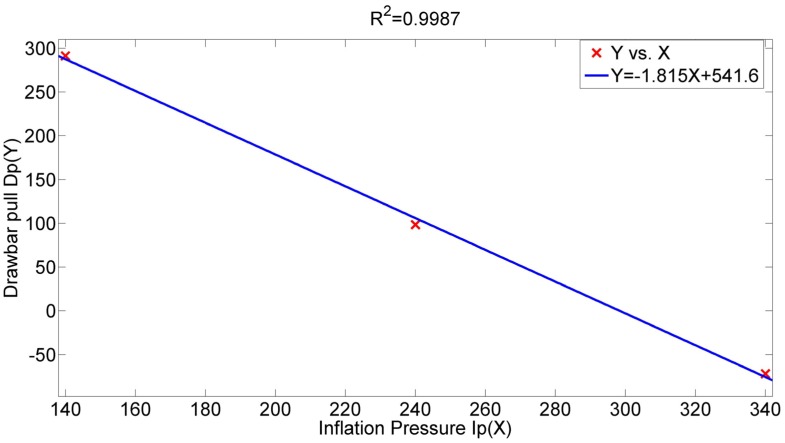
Drawbar pull with respect to inflation pressure.

Figure 5 represents the investigation results of inflation pressure’s effect on drawbar pull at five levels of vertical load (9, 10, 11, 12 and 13 kN) and six levels of slip ratio (0.1, 0.2, 0.3, 0.4, 0.5 and 0.6). Figure 6 illustrates the relation between inflation pressure and drawbar pull (vertical load is set to 13 kN and slip ratio 0.5). Therefore a general rule is indicated that drawbar pull obviously decreases with the increase of inflation pressure and the relation tends to be a linear relationship. Meanwhile, when the slip ratio is set to 0.3 and the inflation pressure increases from 140 to 340 kPa, the drawbar pull decreases form 571.5 to 345.7 N with the vertical load at level of 9 kN and decreases form 243.3 to −115.0 N with the vertical load at 13 kN as shown in Figure 5c. The corresponding decreasing rates are 39.51% and 147.3%. It can be inferred that drawbar pull tends to have a more distinct decrease at larger vertical load, which verifies the combination effect of inflation pressure and vertical load.

#### 2.3.4. Effect of Slip Ratio on Drawbar Pull

**Figure 7:**
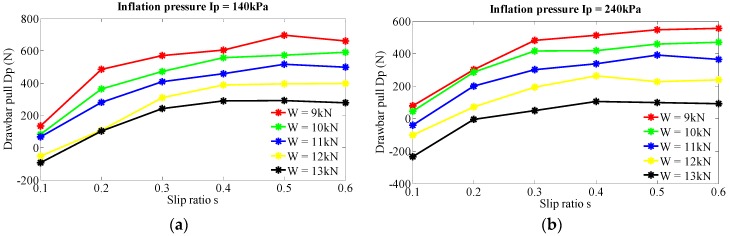

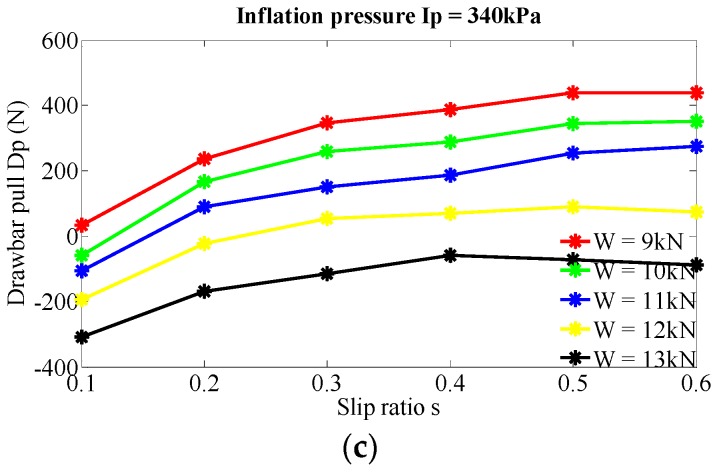
Effect of slip ratio on the drawbar pull at various levels of vertical load and inflation pressure. (**a**) Inflation pressure Ip = 140 kPa; (**b**) Inflation pressure Ip = 240 kPa; (**c**) Inflation pressure Ip = 340 kPa.

**Figure 8:**
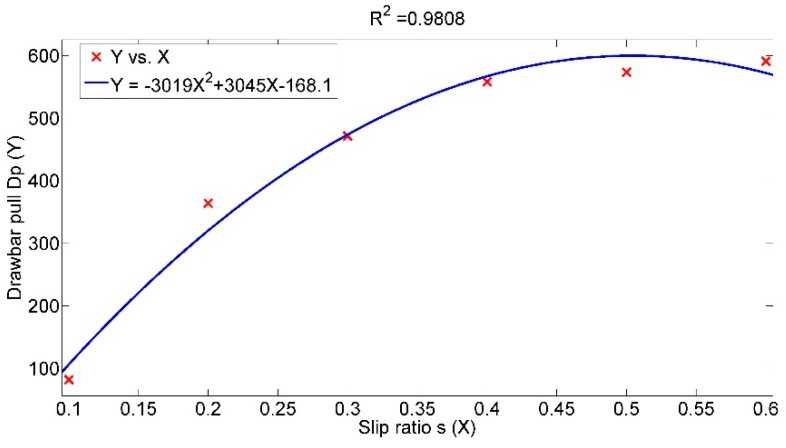
Drawbar pull with respect to slip ratio.

Wheel slip ratio is an important state variable during vehicle movement. Almost all the traction performance-related indexes are affected by slip ratio. Except for inherent vehicle characteristics and terrain parameters, slip ratio is also a reflection of the manner of operation. Investigation of slip ratio’s effect on drawbar pull can provide an instruction for the real-time operation of military vehicles. Figure 7 illustrates the analysis results of slip ratio’s effect on drawbar pull. It is obvious that drawbar pull is an increasing function of slip ratio. From the increasing pattern of the curves, two distinct phases of slip ratio are indicated. When the slip ratio is in the range of 0–0.3, the drawbar pull increases rapidly. This phase can be defined as the rapid growth phase. For the range of 0.3–1, large variation of the slip ratio causes relatively slight change of the drawbar pull, which is defined as the steady growth phase. When vertical load is set to 10kN and inflation pressure 140 kPa, Figure 8 give the relation between slip ratio and drawbar pull and an approximate quadratic regression relationship is indicated, so it can be inferred that slip ratio is an effective influencing factor of drawbar pull and their relation is also complicated.

In conclusion, through specific experimental analysis based on real vehicle test data, vertical load, inflation pressure, and wheel slip ratio are confirmed as the effective factors that influence drawbar pull. The experimental analysis can be considered as a further validation of previous researchers’ findings, and the results come with a high degree of consistency. The relations between drawbar pull and its influencing factors are nonlinear and complex. Hence, urgent demand is elicited for effective new models to accurately predict drawbar pull. The following section presents the proposed novel model based on improved relevance vector machine.

## 3. Methodology

### 3.1. Relevance Vector Machine

Tipping proposed the RVM to recast the main ideas behind support vector machine (SVM) in a Bayesian context, and using mechanisms similar to Gaussian processes [25]. A brief review of Tipping’s paper is presented here for a brief description of RVM. As a supervised learning, RVM starts with a set of data input ${\left\{x_{n}\right\}}_{n-1}^{N}$ and their corresponding target vector ${\left\{t_{n}\right\}}_{n-1}^{N}$. The aim of this training set is to learn a model of the dependency of the target vectors on the inputs in order to make accurate prediction of t for previously unseen value of x. The prediction is estimated based on a function of the following form:

(1) $$t(x)=\sum_{i=1}^{N}w_{i}k(x,x_{i})+w_{0}+\varepsilon_{n}$$

where w = {w_1_, w_2_, …, w_N_} is the weight vectors, w_0_ is bias and k(x, x_i_) is a kernel function, ε_n_ = N(0, σ_2_) is a zero mean Gaussian process.

The likelihood of the complete data set can be written as:

(2) $$p(t|w,\sigma^{2})={\left(2\pi \sigma^{2}\right)}^{-N/2}exp\{-\frac{1}{2\sigma^{2}}\|t-\Phi w\|\}$$

where Ф(x_i_) = [1, k(x_i_, x_1_), k(x_i_,x_2_),…, k(x_i_, x_N_)]^T^.

Without imposing the hyperparameters on the weights, the maximum likelihood of Equation (2) will suffer from sever overfitting. Therefore, Tipping recommended imposition of some prior constrains on the parameters w by adding a complexity to the likelihood or error function. This priori information controls the generalization ability of the learning process. An explicit zero-mean Gaussian prior probability distribution over theweights, w, with diagonal covariance of α is proposedas follows:

(3) $$p(w|\alpha )=\prod_{i=0}^{N}N(w_{i}|0,{\alpha_{i}}^{-1})$$

where α is a vector of N + 1 hyperparameters.

Consequently, by using Baye’s rule, the posterior overall unknowns could be computed, given the defined non-informative prior-distributions:

(4) $$p(w,\alpha ,\sigma^{2}|t)=\frac{p(t|w,\alpha ,\sigma^{2})p(w,\alpha ,\sigma )}{\int p(tw,\alpha ,\sigma^{2})p(w,\alpha ,\sigma^{2})dwd\alpha d\sigma^{2}}$$

However, we cannot calculate the posterior from Equation (4) directly because we cannot perform the normalizing integral involved. Instead, Tipping suggested the decomposition of the posterior according to Equation (5) to facilitate the solution. Then the posterior distribution over the weights is given as Equation (6):

(5) $$p(w,\alpha ,\sigma^{2}|t)=p(w|t,\alpha ,\sigma^{2})p(\alpha ,\sigma^{2}|t)$$

(6) $$p(w|t,\alpha ,\sigma^{2})=\frac{p(t|w,\sigma^{2})p(w|\alpha )}{p(t|\alpha ,\sigma^{2})}={\left(2\pi \right)}^{-(N+1)/2}{\left|\Sigma \right|}^{-1/2}\exp\{-\frac{1}{2}{\left(w-\mu \right)}^{T}\Sigma^{-1}(w-\mu )\}$$

where the posterior covariance and mean are respectively:

(7) $$\Sigma ={\left(\sigma^{-2}\Phi^{T}\Phi +A\right)}^{-1}$$

(8) $$\mu =\sigma^{-2}\Sigma \Phi^{T}t$$

In which A = (α_0_, α_1_, …, α_N_). Therefore, machine learning becomes a search for the hyperparameters posterior most probable. Predictions for a new data are then made according to integration of the weights to obtain the marginal likelihood for the hyperparameters:

(9) $$p(t|\alpha ,\sigma^{2})={\left(2\pi \right)}^{-N/2}{\left|\sigma^{2}I+\Phi A^{-1}\Phi^{T}\right|}^{-1/2}\times exp\{-\frac{1}{2}t^{T}{\left(\sigma^{2}I+\Phi A^{-1}\Phi^{T}\right)}^{-1}t\}$$

### 3.2. Multiple-Kernel RVM

As different kernel functions can produce different RVM regression functions which can determine the prediction performance of RVM, it is very important to choose a suitable kernel function. Generally, users will employ prior knowledge to select a kernel function from a set of standard kernels, such as the Polynomial kernel, Gaussian kernel and Sigmoid kernel. Nevertheless, a single kernel may not always be suitable, especially for complex regression problems.

To solve thisproblem, multiple kernel learning (MKL) methods have been proposed by researchers [17], which improve the prediction performance through kernel combination. In this study, two kinds of kernels including local kernel and global kernel are employed to construct the regression function of RVM. A typical local kernel is the Gaussian kernel and a typical global kernel is the polynomial kernel (a quadratic polynomial), which can be defined as follows:

(10) $$k_{Gauss}(x,x_{i})=exp\left(-\frac{{\left\|x-x_{i}\right\|}^{2}}{{\sigma_{1}}^{2}}\right)$$

(11) $$k_{Poly}(x,x_{i})={\left(x\cdot {\left(\frac{x_{i}}{{\sigma_{2}}^{2}}\right)}^{T}+1\right)}^{2}$$

where σ_1_ denotes the kernel parameter of the Gaussian kernel and σ_2_ denotes the kernel parameter of the polynomial kernel.

In order to improve the generalization ability of RVM, a multiple-kernel RVM(MkRVM) is constructed by the local kernel function (Gaussian kernel) and global kernel function (polynomial kernel). The proportions of the Gaussian kernel and polynomial kernel are determined by the control parameter λ (0 ≤ λ ≤ 1). Thus, the multiple-kernel function (k_mk_) can be defined as follows:

(12) $$k_{mk}(x,x_{i})=\lambda k_{Gauss}(x,x_{i})+(1-\lambda )k_{Poly}(x,x_{i})$$

Through this linear combination, the multiple-kernel function can inherit all the characteristics of independent kernels and can improve the integral performance of RVM in theory. It will be validated in later part.

### 3.3. Parameter Optimization of RVM Based on PSO

Before training the multiple-kernel RVM model, the kernel width parameter (σ_1_ and σ_2_) and control parameter (λ) should be predetermined, which has great impact on the regression accuracy. In essence, it can be treated as a parameter optimization problem. Particle Swarm Optimization (PSO) has been successfully used in the parameter optimization problem of SVM and RVM [26,27]. Especially, it shows great advantages in the issue of multiple parameters optimization. Recently, intelligent optimization techniques have been applied to the original PSO to improve its performance [28,29,30,31,32,33]. In this paper, nonlinear decreasinginertia weight PSO (NDIWPSO) is employed.

PSO uses a swarm of particles that are updated from iteration to iteration. Here, the particle is composed of the kernel width parameters and the control parameter. The position of each particle represents a possible solution, and the optimal solutionis searched by continually updating the velocity and position. Each particle moves in the direction determined by its previously best local position and its best global position. The velocity and position are updated according to the following equations.

(13) $$v_{i}(t+1)=\omega_{v}(t)\cdot v_{i}(t)+c_{1}\cdot rand_{1}\cdot (pbest_{i}(t)-p_{i}(t))+c_{2}\cdot rand_{2}\cdot (gbest_{i}(t)-p_{i}(t))$$

(14) $$p_{i}(t+1)=p_{i}(t)+\beta \cdot v_{i}(t+1)$$

in which, v_i_ and p_i_ represent the velocity and position of the particle i, respectively; pbest_i_ is the best position of the particle i and gbest_i_ is the global best position of the swarm; c_1_ and c_2_ denote two positive acceleration constants for regulating the relative velocities and they are usually set to 1.5; rand_1_ and rand_2_ represent random variables in the range [0,1]; β is a constraint factor used to control the velocity weight, which is usually set to 1. The inertia weight ω_v_ is an important factor in PSO technique, which is a user-defined parameter.Together with c_1_ and c_2_, it controls the contribution of past velocity values to the current velocity of the particle. A large inertia weight biases the search toward global exploration, while a smaller inertia weight directs toward fine-tuning the current solutions (exploitation).Suitable selection of the inertia weight can provide a balance between the global and the local search [34]. It is considered as a constant value in original PSO. In NDIWPSO, it is defined as in Equation (15) to balance the global and local exploitation capability. ω_max_ is the maximum inertia weight and ω_min_ is the minimum inertia weight; H is the maximum iteration:

(15) $$\omega_{v}(t)=\omega_{\max}-\left((\omega_{\max}-\omega_{\min})/\sqrt{H-1}\right)\sqrt{t-1}$$

We perform the optimization procedure over all the training samples to obtain a most excellent generalization performance of the regression model. Root mean squared error (RMSE) of all training samples is used to evaluate the performance of the RVM models with the different particles and it is called the fitness function as in Equation (16). y_act_ is the actual value and y_pre_ is the prediction value; N is the number of the training set. In the process of implementation, leave-one-out cross validation (LOO) is employed:

(16) $$Fitness(RMSE)={\left(\frac{1}{N}\sum_{i=1}^{N}{\left(y_{pre,i}-y_{act,i}\right)}^{2}\right)}^{1/2}$$

**Figure 9:**
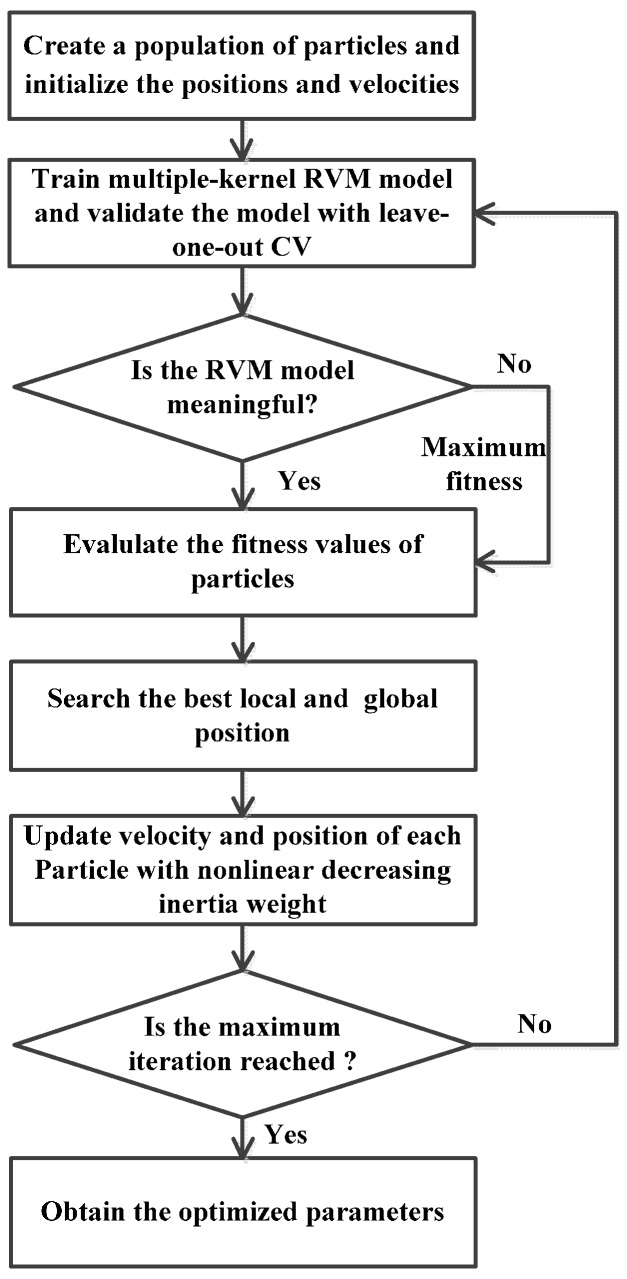
Process of optimizing the RVM parameters with NDIWPSO.

Figure 9 is the flow chart of optimizing the RVM parameters with NDIWPSO and the implementation process can be described as follows:

*Step 1*. Initialize the swarm size, maximum of iterations and the velocity and position for each particle.

*Step 2*. Train the multiple-kernel RVM, and evaluate the fitness of each particle through the method of leave-one-out cross validation. It is worth pointing out that not all the positions of each particle can make the RVM model meaningful. When the situation happens, it means that the position is not a possible solution, and here the corresponding fitness is set to a large enough value to ensure the accomplishment of the whole optimization procedure.

*Step 3*. Update the best position of each particle and the global position of the swarm according to the fitness evaluation results by Equation (16).

*Step 4*. Update the velocity and position of each particle by Equations (13)–(15), respectively.

*Step 5*. The same procedures from Step 2 to Step 4 are repeated until the maximum iteration is reached.

Once the optimized parameters (σ_1_, σ_2_ and λ) are obtained, the multiple-kernel RVM model can be established.

### 3.4. Satisfactory Criteria

To evaluate the performance of new models more comprehensively, mean absolute percentage error (MAPE) and coefficient of determination (R^2^) are also employed as additional satisfactory criteria in this study. R^2^ are given by the following equations:

(17) $$R^{2}={\left(\frac{Cov(y_{act},y_{pre})}{\sigma_{y_{act}}\sigma_{y_{pre}}}\right)}^{2},Cov(y_{act},y_{pre})=\frac{1}{N}\sum_{i=1}^{N}\left(y_{act,i}-\bar{y_{act}})(y_{pre,i}-\bar{y_{pre}}\right)$$

where R^2^ is squared correlation coefficient and Cov(y_act_, y_pre_) is covariance between actual and predicted values. In addition, y_act_ and y_pre_ denote the average result of actual and predicted value. Moreover, σ is the relevant standard deviation. A higher correlation value expresses a better prediction performance.

## 4. Results and Discussion

**Figure 10:**
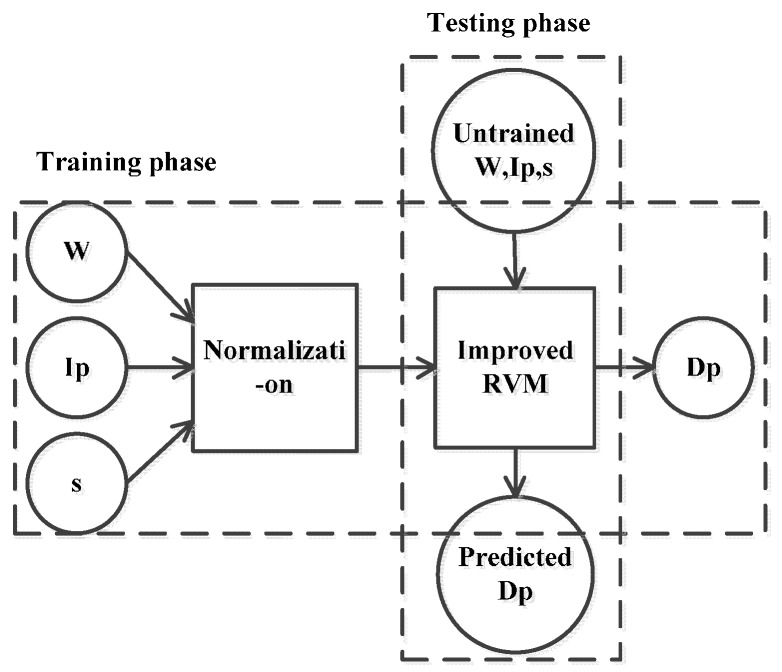
Schematic overview of the proposed RVM model.

Drawbar pull is a primary index in the evaluation of military vehicle trafficability on soft terrain. It is of immediate significance to predict drawbar pull accurately. Through the detailed experimental analysis based on real vehicle test data, vertical load, inflation pressure and slip ratio are confirmed as effective influencing factors while moving velocity’ effect on drawbar pull can be ignored. A linear or quadratic regression relationship is verified between drawbar pull and its effective influencing factors. Influencing factors’combination effect is also inferred. Meanwhile, vertical load, inflation pressure and slip ratio can be considered as independent variables. Based on this consideration, mathematical statistics methods are suggested as a proper solution for the prediction of drawbar pull. In this study, a novel prediction model of drawbar pull is proposed by combining the improved RVM method as shown in Figure 10. (W, Ip, s) represent the model inputs while Dp is the output. Real vehicle test data is used to train and test the new model. Among all the available raw data sets illustrated in Figure 3, Figure 5 and Figure 7, 80 data sets are prepared for the training phase and the remaining 10 for the testing phase. During the training phase, the RVM model is trained by the training data sets and the model is validated by the testing data sets. The whole procedure is carried out by using MATLAB programs.

The experimental data are normalized to the range of [0,1] in order to improve the generalization ability of the prediction models. We use NDIWPSO to select the kernel parameters (σ_1_, σ_2_) and the control parameter λ. In NDIWPSO, the two positive acceleration constants c_1_ and c_2_ are set to 2; β is set to 0.75; ω_max_ is set to 0.9 and ω_min_ is set to 0.4; the maximum iteration H is set to 200. Then, we set the value range of the three-dimensional particle, σ_1_ of [0.1, 20], σ_2_ of [0.1, 20] and λ of [0,1]. After NDIWPSO, the optimal parameters of the multiple-kernel RVM model are obtained with σ_1_ = 2.456, σ_2_ = 14.96 and λ = 0.6808. As the Gaussian kernel RVMmodel (GaussRVM) and the polynomial kernel RVM model (PolyRVM) are utilized to compare with the MkRVM model, their kernel parameters are also optimized by NDIWPSO (σ_1_ = 3.206 and σ_2_ = 8.264).

**Figure 11:**
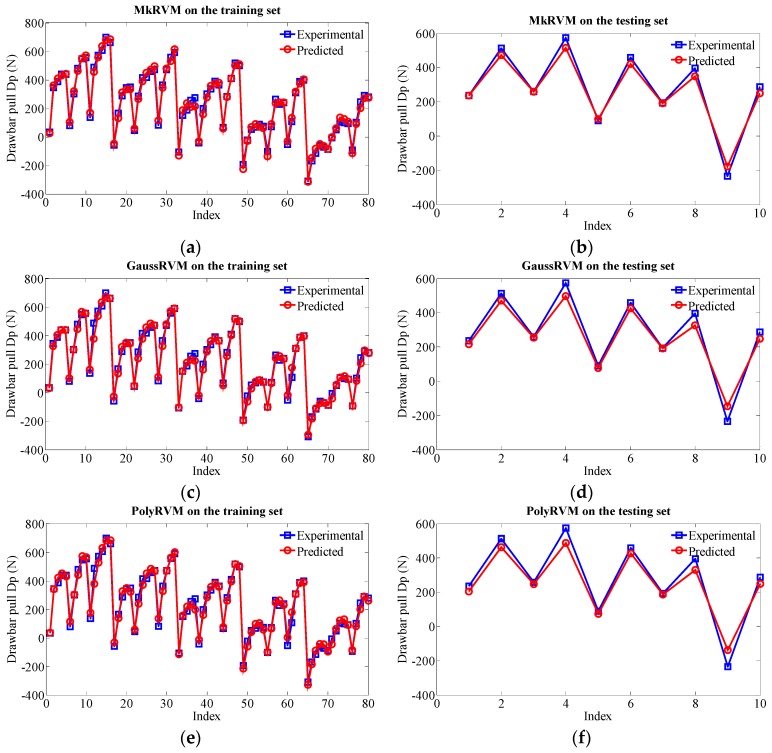
Prediction results of the three RVM model with three kernel functions on the training set and the testing set. (**a**) MkRVM on the training set; (**b**) MkRVM on the testing set; (**c**) GaussRVM on the training set; (**d**) GaussRVM on the testing set; (**e**) PolyRVM on the training set; (**f**) PolyRVM on the testing set.

**Figure 12:**
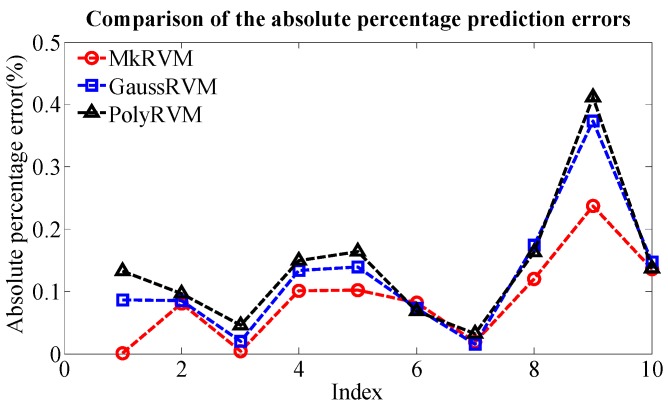
The comparison of the absolute percentage predictionerrors among the MkRVM, GaussRVM and PolyRVM.

**Figure 13:**
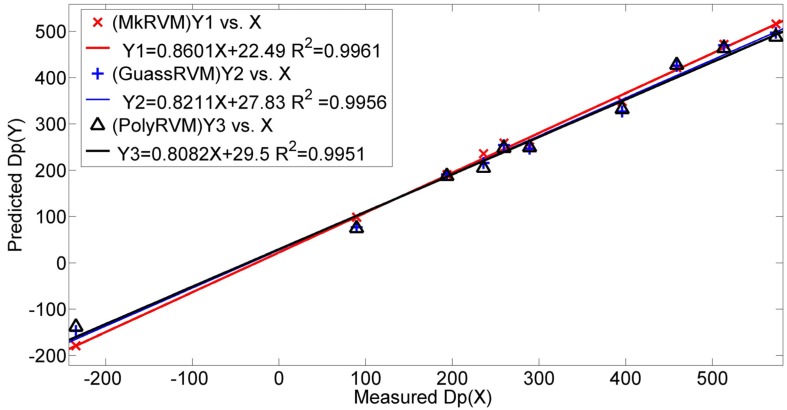
The comparison of correlation coefficient between measured Dp and predicted Dp from the MkRVM, GaussRVM and PolyRVM.

Figure 11 represents the prediction results of the three RVM models, the MkRVM, GaussRVM and PolyRVM, respectively. In each subfigure, we set the index of dataset as the abscissa and set the output parameter (Dp) as the ordinate. Figure 11a,c,e denote the validation result on the training set while Figure 11b,d,f refer to the prediction performance on the testing set. Figure 12 gives the comparison of the absolute percentage prediction errors among the three RVM models. Figure 13 illustrates the comparison of correlation coefficient between measured Dp and predicted Dp. The more detailed numerical comparison is shown in Table 2. It is inferred that the MkRVM outperforms the GaussRVM and PolyRVM no matter on the training set or on the testing set. On the testing set, the MkRVM gains the smallest MAPE of 9.023%, the minimum RMSE of 37.29 N and the optimal R^2^ of 0.9961. It is a good validation of the MkRVM’s outstanding generalization ability and prediction capacity, which is consistent with the theory. Comparatively speaking, the GaussRVM has a better performance than the PolyRVM with the MAPE of 12.44%, RMSE of 46.64 N and R^2^ of 0.9956 on the testing set. The value of the control parameter λ (0.6808) also shows a reflection of this point.

**Figure 14:**
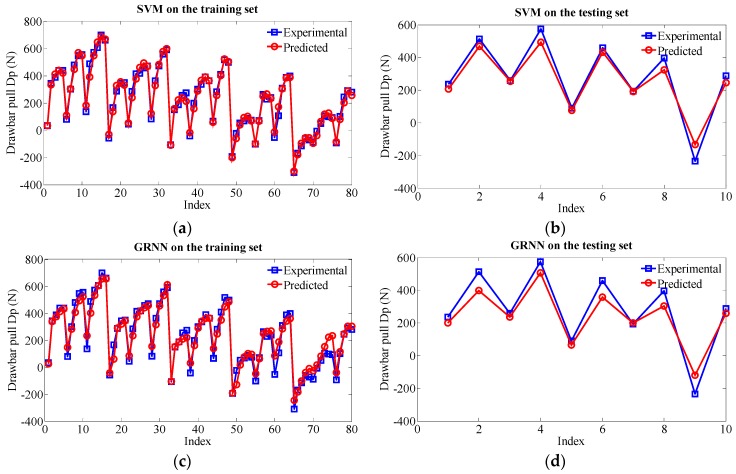
Prediction results of the SVM model and GRNN model on the training set and the testing set. (**a**) SVM on the training set; (**b**) SVM on the testing set; (**c**) GRNN on the training set; (**d**) GRNN on the testing set.

**Figure 15:**
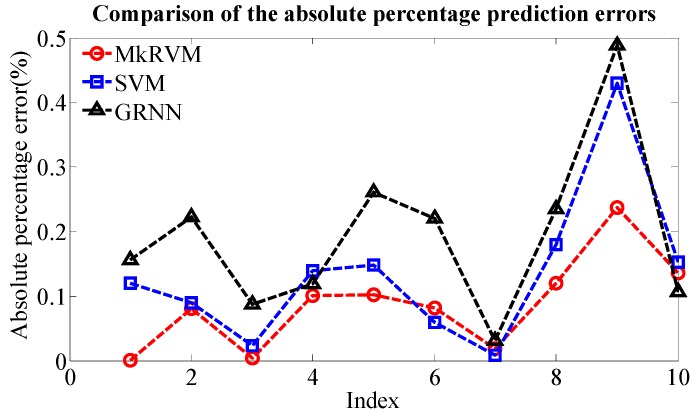
The comparison of the absolute percentage prediction errors among the MkRVM, SVM and GRNN.

**Figure 16:**
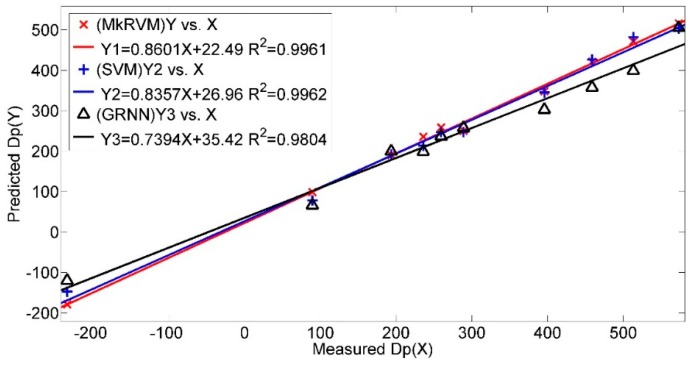
The comparison of correlation coefficient between measured Dp and predicted Dp from the MkRVM, SVM and GRNN.

In order to cross-validate the proposed RVM model, a comparison is made among the RVM model, a support vector machine (SVM) model and a generalized regression neural network (GRNN) model. To train the SVM model, the kernel of RBF is adopted (the Gaussian kernel). As implementation, the open-source library LIBSVM is adopted [35]. We should emphasize the necessity of careful selection of SVM model parameters to obtain high accuracy. Three main SVM hyperparameters are of great significance. c (cost) is defined as a penalty parameter, g points to the setup of gamma in SVM kernels and p denotes a parameter of the insensitive loss function. The NDIWPSO is also used for the parameter optimization of the SVM model. Moreover, the k-fold cross validation method (KCV, k = 5) and leave-one-out cross validation method (LOO) are used to optimize the hyperparameters c and g [36]. Among the optimization results, the smaller c obtained by KCV is selected as the optimal one (c = 6.964, g = 0.4543). For the epsilon-insensitive parameter p, we made an investigation in the range of [10^−1^, 10^−6^]. When p is set to 10^−3^, the SVM model has a better prediction performance. For the implementation of the GRNN model, a cross validation method is utilized to find the best relevant parameter, Spread, with the result of 0.4. Figure 14a,b demonstrate the prediction results of SVM on the training set and testing set, respectively. Figure 14c,d give the prediction results of GRNN. The comparison of the absolute percentage prediction errors among the MkRVM, SVM and GRNN is shown in Figure 15 and the comparison of correlation coefficient between measured Dp and predicted Dp is displayed in Figure 16. The detailed numerical comparison is listed in Table 3. From the results, the SVM model shows a quite good performance while the GRNN method gains non-ignorable prediction errors. The SVM model outperforms the GaussRVM and PolyRVM with the MAPE of 11.71%, RMSE of 44.21N and R^2^ 0f 0.9962 on the testing set. Although the SVM model performs equal to the MkRVM on R^2^, it gains higher MAPE and larger RMSE. As such, the MkRVM can still be considered as the most outstanding model for its comprehensive performance.

For a comprehensive evaluation of different models’ prediction performance, a comparison is made in terms of computation efficiency as listed in Table 4. The simulation was performed using Matlab code on a 2.13 G Inter Core 2 PC with 1.89 G RAM. In the process of parameter optimization, the three RVM models take much more time than the SVM and GRNN. In particular, the MkRVM takes 342.7 s to confirm the suitable parameters. In the process of model training, the SVM model outperforms the RVM models and the GRNN model. In the process of model testing, the RVM models have a better performance than the SVM and GRNN model. In general, the MkRVM has relatively bad computation efficiency in the process of model training including parameter optimization although it performs well in the process of model testing, so, the MkRVM doesn’t show an absolute advantage in terms of computation efficiency. However, considering that this study is intended for offline usage, the real-time requirements are not so high and the emphysis is laid on the terms of prediction accuracy. From this perspective, the MkRVM can still be considered as an effective model.

**Figure 17:**
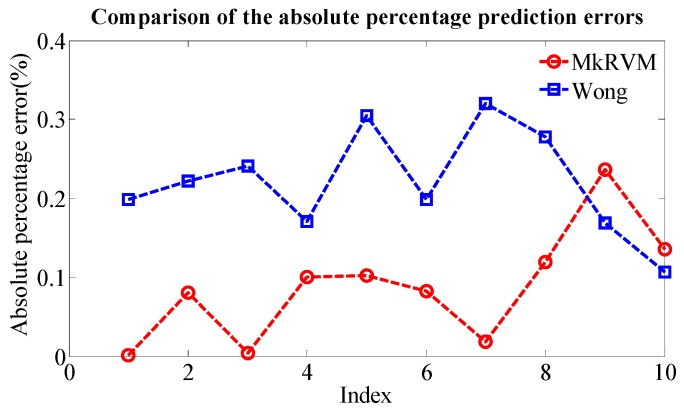
The comparison of the absolute percentage prediction errors between the MkRVM and Wong’s model.

**Figure 18:**
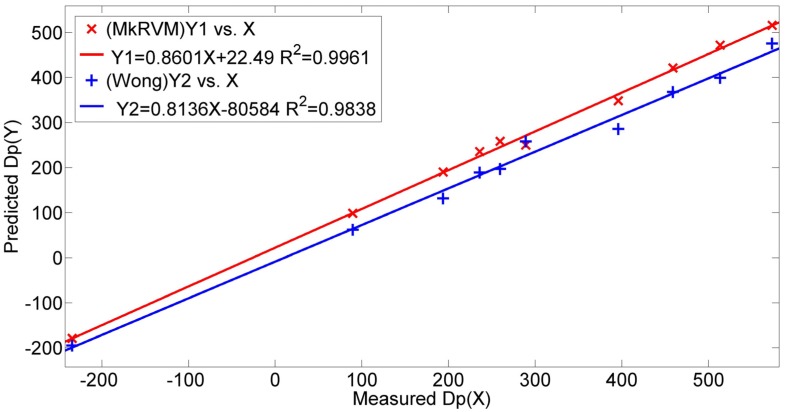
The comparison of correlation coefficient between measured Dp and predicted Dp from the MkRVM and Wong’s model.

For a further validation of the capability of the proposed novel model, a comparison is made between the MkRVM model and the traditional prediction model of drawbar pull. Wong’s straight line model is imposed to predict drawbar pull [1]. The simplified model can be found in [24]. Various vehicular parameters and terrain parameters are involved as listed in Table 5. k_c_, k_Φ_ and n are the pressure-sinkage coefficients of the terrain; c and Φ denote the cohesion stress and internal friction angle of soil while K denotes the shear deformation modulus. These terrain parameters are measured by specific soil tests in laboratory with the soil collected in field tests. The experimental procedure is similar to that found in [37]. D represents the wheel diameter and b refers to the width of the wheel-soil contact patch. Another necessary parameter, wheel sinkage (Z) is estimated by Lyasko’s model [38]. It can be seen that the modeling and predicting process are quite complicated. Table 6 demonstrates the comparison results of the MkRVM model and Wong’s model. Figure 17 gives the comparison of the absolute percentage prediction errors while Figure 18 illustrates the comparison of correlation coefficient between measured and predicted Dp. On the testing set, the prediction accuracy of Wong’s model is unsatisfactory with the MAPE of 23.91%, RMSE of 61.54 N and R^2^ of 0.9838. It can be concluded that the proposed RVM model greatly improves the prediction accuracy comparing to the traditional model. What’s more, it reduces the complexity in the predicting process.

## 5. Conclutions

This paper addresses the issue of prediction of a military vehicle’s drawbar pull. The proposed novel model is based on an improved RVM. The multiple kernel learning method is used to improve its prediction performance while nonlinear decreasinginertia weight particle swarm optimization is employed to optimize the kernel parameters and control parameter involved. Before training the multiple-kernel RVM model (MkRVM), a series of experimental analyses are done to investigate the relation between drawbar pull and its influencing factors. Vertical load, inflation pressure and slip ratio are confirmed as the effective influencing factors and their relations are complex and nonlinear. Real vehicle test data lays the foundation for the experimental analysis and model validation. Through the comparison of the prediction performance among the MkRVM model, the GaussRVM model, the PolyRVM model, the SVM model and the GRNN model, it is indicated that the MkRVM is a preferable model in this case. Finally, through the comparison to the traditional prediction model of drawbar pull, it can be inferred the proposed model significantly improves the prediction accuracy.

The great potential of mathematical statistics methods is indicated in further researches of wheel-interactions. Three effective influencing factors of drawbar pull are used to establish the novel model at present, and more influencing factors, for example the terrain parameter (cone index), are to be included for a more integrated prediction model. Moreover, there still exist non-ignorable prediction errors in the proposed model. The MkRVM model is a preferable model at present and more work will be done for better solutions.

## Figures and Tables

**Table 1:** Summary of the adopted levels of various factors.

Velocity V (m/s)	Vertical Load W (kN)	Inflation Pressure Ip (kPa)	Slip Ratio s
1.8	8 + 1	140	0.1
3.6	8 + 2	240	0.2
5.4	8 + 3	340	0.3
7.2	8 + 4	-	0.4
-	8 + 5	-	0.5
-	-	-	0.6

**Table 2:** Comparison of the prediction performance of different kernel RVM models.

RVM Kernels	Training Set			Testing Set		
	MAPE	RMSE(N)	R^2^	MAPE	RMSE(N)	R^2^
Mk	14.70%	22.94	0.9948	9.023%	37.29	0.9961
Gauss	19.96%	25.01	0.9940	12.44%	46.64	0.9956
Poly	24.99%	29.34	0.9916	13.97%	52.03	0.9951

**Table 3:** Comparison of the prediction performance among the MkRVM, SVM and GRNN.

Models	Training Set			Testing Set		
	MAPE	RMSE(N)	R^2^	MAPE	RMSE(N)	R^2^
**MkRVM**	14.70%	22.94	0.9948	9.023%	37.29	0.9961
**SVM**	17.43%	24.08	0.9944	11.71%	44.21	0.9962
**GRNN**	48.91%	49.50	0.9785	37.22%	73.52	0.9804

**Table 4:** Comparison of different models’ computation efficiency.

Models	MkRVM	GaussRVM	PolyRVM	SVM	GRNN
**Parameteroptimization**	342.7 s	169.33 s	181.14 s	25.65 s	2.945 s
**Training**	0.0607 s	0.0667 s	0.0522 s	0.0092 s	0.0295 s
**Testing**	0.000088 s	0.000059 s	0.000081 s	0.00045 s	0.0038 s

**Table 5:** Parameters involved in the traditional semi-empirical model.

Terrain Parameters						Vehicular Parameters	
c(kPa)	Φ(°)	K(m)	n	k_c_(kN/m^n+1^)	k_Φ_(kN/m^n+2^)	D(m)	b(m)
7.58	14	0.025	0.85	43.68	499.3	0.98	0.32

**Table 6:** Comparison of the prediction performance of the RVM model and the traditional model.

Models	Testing Set		
	MAPE	RMSE(N)	R^2^
**RVM**	9.023%	37.29	0.9961
**Wong**	23.91%	61.54	0.9838
